# IL-37 Confers Protection against Mycobacterial Infection Involving Suppressing Inflammation and Modulating T Cell Activation

**DOI:** 10.1371/journal.pone.0169922

**Published:** 2017-01-11

**Authors:** Haipeng Liu, Ruijuan Zheng, Peng Wang, Hua Yang, Xin He, Qun Ji, Wenjuan Bai, Hao Chen, Jianxia Chen, Wenxia Peng, Siyu Liu, Zhonghua Liu, Baoxue Ge

**Affiliations:** 1 Shanghai TB Key Laboratory, Shanghai Pulmonary Hospital, Tongji University, Shanghai, China; 2 Department of Microbiology and Immunology, Tongji University School of Medicine, Shanghai, China; 3 Clinical Translational Research Center, Shanghai Pulmonary Hospital, Tongji University, Shanghai, China; 4 Department of TB, Shanghai Pulmonary Hospital, Tongji University, Shanghai, China; Fundació Institut d’Investigació en Ciències de la Salut Germans Trias i Pujol, Universitat Autònoma de Barcelona, SPAIN

## Abstract

Interleukin-37 (IL-37), a novel member of the IL-1 family, plays fundamental immunosuppressive roles by broadly reducing both innate inflammation and acquired immunity, but whether it is involved in the pathogenesis of tuberculosis (TB) has not been clearly elucidated. In this study, single nucleotide polymorphism (SNP) analysis demonstrated an association of the genetic variant rs3811047 of *IL-37* with TB susceptibility. In line with previous report, a significant elevated IL-37 abundance in the sera and increased expression of IL-37 protein in the peripheral blood mononuclear cells (PBMC) were observed in TB patients in comparison to healthy controls. Moreover, release of IL-37 were detected in either macrophages infected with *Mycobacterium tuberculosis* (Mtb) or the lung of BCG-infected mice, concurrent with reduced production of proinflammatory cytokines including IL-6 and TNF-α. Furthermore, in contrast to wild-type mice, BCG-infected IL-37-Tg mice manifested with reduced mycobacterial burden and tissue damage in the lung, accompanied by higher frequency of Th1 cell and less frequencies of regulatory T cells and Th17 cells in the spleen. Taken together, our findings demonstrated that IL-37 conferred resistance to Mtb infection possibly involving suppressing detrimental inflammation and modulating T cell responses. These findings implicated that IL-37 may be employed as a new molecular target for the therapy and diagnosis of TB.

## Introduction

Tuberculosis (TB), a leading infectious disease caused by the bacterial pathogen *Mycobacterium tuberculosis* (Mtb), remains a major threat to public health and a frequent cause of morbidity and mortality globally (WHO, WHO Global Tuberculosis Report 2016). Following mycobacterial infection, multiple arms of host immune system are mobilized to defense the invasion of mycobacteria [1]. Conserved mechanisms employed by host for the recognition of Mtb are essential for the containment of bacterial infection in the early stage and instruction of specific adaptive immunity [1–5]. A delicate balance between protective immunity and destructive pathology shapes the lung environment and directs granuloma development, which eventually decides the occurrence of TB [6,7].

Non-resolving inflammation is closely correlated with the pathologic consequences of TB [6,7]. Cytokines are crucial for the initiation, progression, and resolution of inflammation [8]. Macrophages serve as habitat of mycobacteria and are a major source of cytokines involved in mycobacterial control [9]. *Mycobacterium tuberculosis* (Mtb)-infected macrophages and monocytes produce proinflammatory cytokines including IL-1, IL-6, tumor necrosis factor (TNF)-α [10]. Differing from other members of the IL-1 family which are proinflammatory, interleukin-37 (IL-37) mainly exerts anti-inflammatory effects by reducing both innate and acquired immunity [11,12]. It has been documented that IL-37 was induced in human type II alveolar epithelial cells in response to mannose-capped lipoarabinomannan (ManLAM), an immunosuppressive epitope of Mtb [13]. In addition, IL-37 level was reported to be elevated in TB patients and correlate with levels of cytokines including interferon (IFN) γ, IL-12, IL-10 and transforming growth factor (TGF) β. It was implicated to be involved in the process of Mtb infection by reducing proinflammatory cytokines and skewing macrophages towards an M2-like phenotype [14]. However, the exact role of IL-37 in the pathogenesis of TB is not defined. Because a complete open reading frame (ORF) of IL-37 homolog is lacking in the mice, we generated a strain of transgenic mice expressing human *IL-37b*, designated IL-37-Tg mice. In this study, we employed both macrophage infection model and mouse intranasal BCG infection model to dissect the role of IL-37 in the process of Mtb infection. We demonstrated that IL-37 conferred protection against Mtb infection possibly involving suppressing detrimental inflammation and promoting Th1 cell activation. These findings indicated that IL-37 may be employed as a novel molecular target for the therapy and diagnosis of TB.

## Results

### Association of *IL-37* genetic variant rs3811047 with susceptibility to pulmonary TB (PTB)

Cytokines production in response to Mtb infection is critical for pathogen control by mediating innate immunity and subsequent shaping of adaptive immune response [8,10]. Since IL-37 was reported to be a fundamental inhibitory cytokine [12], we explored the association of the polymorphism of this gene with PTB susceptibility. The allelic frequencies of 3 SNPs among *IL-37* were analyzed between 435 PTB and 375 healthy donors. The minor allele frequencies of all 3 SNPs were >1% and were in Hardy-Weinberg equilibrium in control group **(data not shown)**. However, only the allelic frequency of rs3811047 was significantly different between PTB and healthy controls (*p* = 0.018) (Table 1). We further compared the genotype frequencies of *IL-37* gene in PTB and the controls, and a significant difference was observed for rs3811047 (*p* = 0.002), but not for rs2723176 and rs6717710 (Table 2). Therefore, *IL-37* variant rs3811047 was associated with susceptibility to PTB.

**Table 1:** Allelic distribution and frequencies of *IL-37* in pulmonary tuberculosis patients and controls.

SNP ID	Alleles (MAF/Minor)	Group	No.	Alleles Frequency (%)		*P* Value	OR(95% CI)
rs2723176	C/A	TB	435	588(67.6)	282(32.4)	0.201	1.144(0.931~1.406)
		Controls	374	483(64.6)	265(35.4)		
rs3811047	G/A	TB	410	702(85.6)	118(14.4)	**0.018**	1.381(1.055~1.806)
		Controls	369	599(81.2)	139(18.8)		
rs6717710	T/C	TB	435	697(80.1)	173(19.9)	0.577	0.932(0.727~1.195)
		Controls	370	601(81.2)	139(18.8)		

**Table 2:** Frequencies of IL-37 genotype in pulmonary tuberculosis patients and controls.

db SNP	Genotype	Genotype Frequency (%)		*p* value
		TB patients	Controls	
rs2723176	C/C	207(47.5)	152(40.6)	0.075
	C/A	174(40.0)	179(47.9)	
	A/A	54 (12.5)	43(11.5)	
rs3811047	G/G	297(72.4)	251(68.0)	**0.002**
	G/A	108(26.3)	97(26.3)	
	A/A	5 (1.3)	21 (5.7)	
rs6717710	T/T	273(62.8)	248(67.0)	0.063
	T/C	151 (34.7)	105(28.4)	
	C/C	11 (2.5)	17(4.6)	

### Elevated expression of IL-37 in TB patients

**Fig 1:**
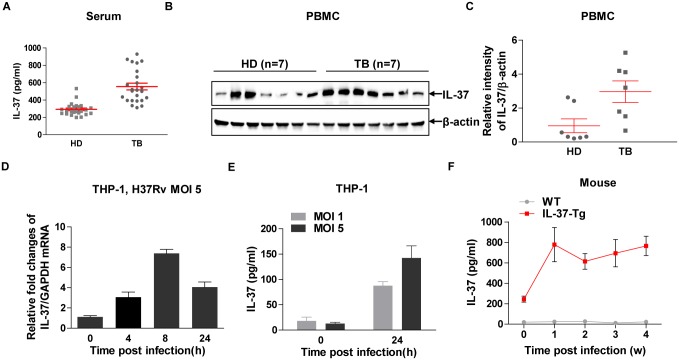
Elevated IL-37 in TB patients and induction of IL-37 in response to Mtb infection. **(A)** ELISA detection the abundance of IL-37 in the sera from healthy donors (HD) (n = 24) and TB patients (TB) (n = 24). Data are mean±SEM. Student’ *t* test was used for statistical analysis. **(B)** Western blot detection of IL-37 in the lysates of PBMC from HD (n = 7) and TB (n = 7). **(C)** Quantification and statistical analysis of the relative density (mean gray value) of IL-37/β-actin as shown in **(B)**. Data are mean±SEM. Student’ *t* test was used for statistical analysis. **(D)** Induction of IL-37 transcripts measured by qRT-PCR in human THP-1 cells infected with H37Rv at MOI 5 for indicated time. **(E)** Secretion of IL-37 protein measured by ELISA in PMA-differentiated human THP-1 cells infected with H37Rv at indicated MOI for 24 h. Data **(D-E)** are mean±SEM and representative of 3 independent experiments. **(F)** Kinetics of IL-37 production measured by ELISA in the lung of WT and IL-37-Tg mice infected with BCG. Results are mean±SEM and are pooled from two independent experiments (n = 7 or 8 for each time point). *, p<0.05; **, p<0.01.

In line with previous report [14], we observed that IL-37 protein was presented in the sera of healthy donors as detected by ELISA (Fig 1A), while its abundance was significantly elevated in active TB patients (Fig 1A). IL-37 exerts its function both intracellularly and extracellularly [12,15], next we detected endogenous expression of IL-37 in the PBMC isolated from both healthy donors (n = 7) and TB patients (n = 7) by Western blot. We observed that IL-37 protein expression was also significantly upregulated in PMBC from TB patients compared with healthy controls (Fig 1B and 1C). Taken together, both production and secretion of IL-37 were markedly enhanced in the peripheral blood of TB patients.

### Mycobacterial infection induces IL-37 expression

In TB, macrophages serve as both habitat and first line of defense against Mtb [9], which are the major source of multiple cytokines and chemokines. To detect whether macrophages produce IL-37 during mycobacteria infection, we infected human monocytic THP-1 cells with virulent Mtb H37Rv and detected the production of IL-37 at the transcriptional and protein level. The results demonstrated that IL-37 transcripts were markedly induced post infection with H37Rv at MOI 5 (Fig 1D). Moreover, IL-37 protein secreted in the supernatants of THP-1 cells markedly increased post infection with H37Rv for 24 h at indicated MOI (Fig 1E). To further detect whether IL-37 expression is responsive to Mtb infection *in vivo*, we generated a strain of transgenic mice expressing human IL-37 designated as IL-37-Tg mouse. Briefly, *IL-37b* gene (RefSeq: NM_014439.3) encoding 218 amino acids was cloned into pBROAD3 vector under the control of ROSA26 promoter (S1 Fig) and electroporated into ES cells. Positive clones were screened and transplanted into receiver mice. The first offsprings were genotyped by PCR (S1 Fig). The expression of IL-37 was further confirmed by Western blot detection of the protein in peritoneal macrophages isolated from the founder mice (S1 Fig). Therefore, we successfully constructed IL-37 transgenic (IL-37-Tg) mice. Then we infected both WT and IL-37-Tg mice with BCG intranasally (i.n.) for indicated times and detected the abundance of IL-37 in the lung by ELISA. We found that IL-37 was highly presented in the lung at all time points examined in the transgenic mice but not the WT mice, and an elevated IL-37 abundance was observed as early as 1 week post infection (Fig 1F). Since the ROSA26 promoter drives gene expression constitutively and ubiquitously, the increased IL-37 in the lung of transgenic mice post infection could be due to increased frequencies and numbers of cells expressing IL-37 including macrophages, dendritic cells and regulatory T cells [11,16]. Overall, recognition of mycobacteria by macrophages led to the induction of IL-37, which may contribute to elevated IL-37 in BCG-infected mice and TB patients.

### Enhanced resistance to mycobacterial infection in IL-37 transgenic mice

**Fig 2:**
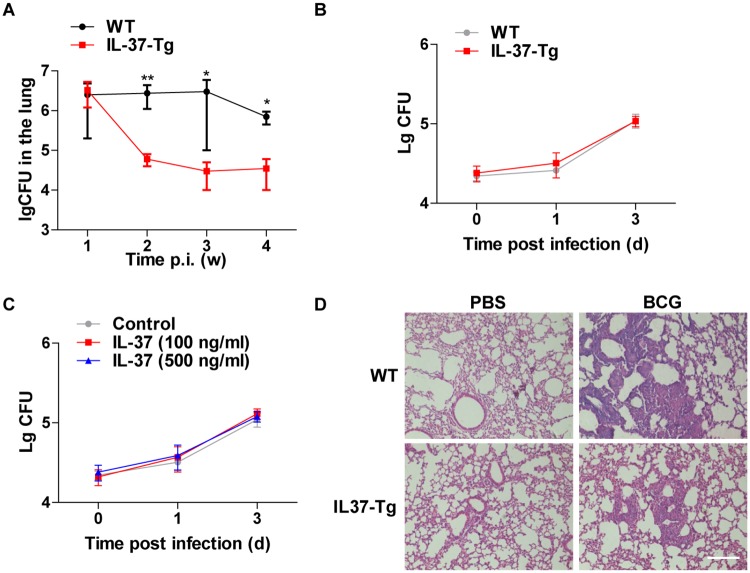
IL-37 protects mice from BCG infection. **(A)** BCG burden in lung, measured in CFUs, in WT mice and IL-37-Tg mice at every week following i.n. infection with 4×10^7^ viable BCG. Data are presented as the median ± the interquartile range (iQR) and are representative of two independent experiments; Mann-Whitney *U* test was used for statistical analysis (n = 4 for each time point). **(B)** Peritoneal macrophages isolated from WT and IL-37-Tg were infected with H37Rv for 1 h at MOI = 5, washed and cultured for indicated time, followed by CFU assay. **(C)** Peritoneal macrophages were infected with H37Rv for 1 h at MOI = 5, washed and cultured in the absence or presence of different concentrations of recombinant IL-37 for indicated time followed by CFU assay. Data **(B-C)** are presented as the median ± the interquartile range (iQR) and representative of three independent experiments, with four duplicates each; Mann-Whitney *U* test was used for statistical analysis. **(D)** Representative H&E staining of lungs from BCG-infected WT (n = 4) and IL-37-Tg mice (n = 4) at 4 weeks p.i. of two independent experiments with comparable results. Scale bar, 100 μm. *, p<0.05; **, p<0.01.

To further characterize the function of IL-37 in the process of mycobacteria infection, we infected both WT and IL-37-Tg mice with *M*. *bovis* BCG intranasally (i.n.) and then assessed bacterial burden in the lung as determined by CFUs every week after infection. The overexpression of IL-37 in the mice led to a significant reduction of bacterial burden from 2 weeks p.i. (Fig 2A). In contrast, comparable bacterial loads were observed in WT and IL-37-Tg macrophages infected with H37Rv (Fig 2B), indicating that IL-37 is dispensable for intracellular growth of mycobacteria in macrophages. In addition, the addition of recombinant human IL-37 in the culture medium didn’t affect the intracellular survival of Mtb (Fig 2C), which suggested that extracellular IL-37 is not involved in regulating the bactericidal effect of macrophages. Therefore, the less bacterial burden in the lungs of IL-37-Tg mice is less likely resulted from enhanced killing activity of macrophages. Compared to WT mice, IL-37-Tg mice also exhibited significant reduction in the infiltration of neutrophil and lymphocyte into the lung at 4 weeks p.i. as measure by H&E staining (Fig 2D), which indicated that IL-37 was critical for reducing overt inflammation and collateral tissue damage. Taken together, we conclude that the presence of IL-37 attenuates mycobacterial infection.

### IL-37 suppresses inflammatory responses in mycobacterial-infected macrophages and mice

**Fig 3:**
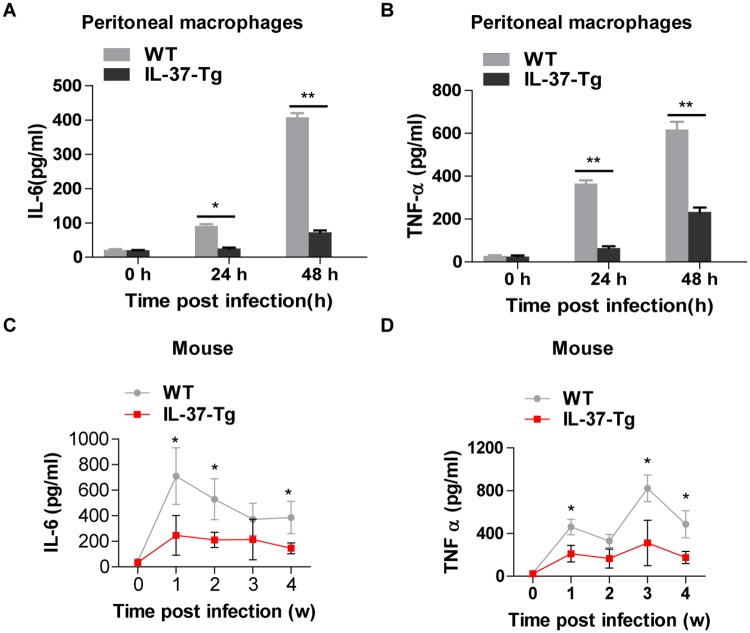
IL-37 suppresses inflammatory responses in mycobacterial-infected macrophages and mice. **(A- B)** ELISA detection of IL-6 **(A)** and TNF-α **(B)** in WT and IL-37-Tg macrophages infected with H37Rv at MOI = 5 for indicated times. Results are the mean±SEM and representative of 3 independent experiments with triplicates each. **(C-D)** Kinetics of IL-6 **(C)** and TNF-α **(D)** production detected by ELISA in the lung of WT and IL-37-Tg mice infected with BCG. Results are the mean±SEM and are pooled from two independent experiments. n = 7 or 8 for each time point; two way ANOVA with Bonferroni’s post test. *, p<0.05; **, p<0.01.

Macrophages are primary target of mycobacteria including Mtb and BCG [17], and cytokines released from macrophages are important mediators of both innate and adaptive immune responses [8,10]. To determine whether IL-37, a fundamental anti-inflammatory cytokine [12], affects mycobacteria-induced cytokine production, we infected WT and IL-37-Tg peritoneal macrophages with H37Rv and then measured the release of IL-6 and TNF-α in the supernatants by ELISA. The overexpression of IL-37 dramatically inhibited H37Rv-induced IL-6 (Fig 3A) and TNF-α (Fig 3B). Next, WT and IL-37-Tg mice were i.n. infected with BCG for indicated times and followed by measurement of the abundance of IL-6 and TNF-α in lung tissue by ELISA. IL-6 and TNF-α protein were induced post BCG infection in both WT and IL-37-Tg mice. However, a marked reduction of both cytokines in IL-37-Tg mice was observed in comparison with WT mice (Fig 3C and 3D). Therefore, IL-37 plays a major role in inhibiting mycobacteria-induced production of proinflammatory cytokines including IL-6 and TNF-α, both *in vitro* and *in vivo*.

### Enhanced Th1 and reduced Treg, Th17 responses in IL-37 transgenic mice during mycobacterial infection

**Fig 4:**
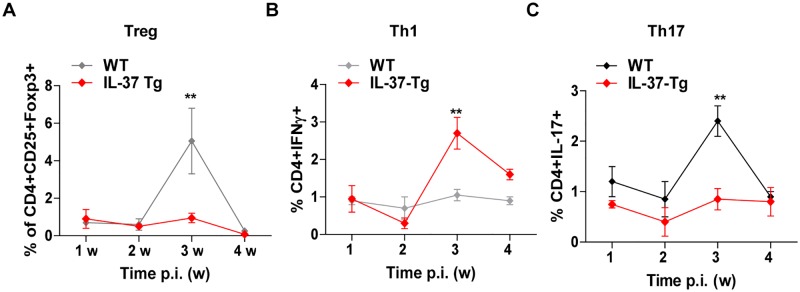
Enhanced Th1 and reduced Treg and Th17 responses in IL-37 transgenic mice during mycobacterial infection. Kinetics of Treg **(A)**, Th1 **(B)** and Th17 **(C)** cells in the spleens of WT and IL-37-Tg mice infected with BCG. The frequencies of CD4^+^CD25^+^FoxP3^+^ **(A)**, CD4^+^IFNγ^+^ **(B)** and CD4^+^IL-17^+^ **(C)** cells among gated lymphocytes were determined at indicated times p.i. Results are the mean±SEM and are pooled from two independent experiments; n = 4 or 5 for each time point; two way ANOVA with Bonferroni’s post test. **, p<0.01.

To determine whether IL-37 affects acquired immunity during BCG infection, we infected mice i.n. with BCG and quantified splenic T cells including Treg, Th1, Th17 cells by flow cytometry over 4 weeks p.i. We observed higher frequency of CD3^+^ T cells in IL-37-Tg mice at 2 weeks post infection with BCG, manifesting with increased frequency of CD4^+^ T cells but not that of CD8^+^ T cells among the CD3+ T cells (S2 Fig). At 3 weeks p.i., IL-37-Tg mice showed higher frequency of CD4^+^IFNγ^+^Th1 cells but lower frequencies of CD4^+^CD25^+^Foxp3^+^Treg cells and CD4^+^IL-17^+^Th17 cells than control animals (Fig 4A–4C). Therefore, IL-37 may be involved in the differentiation of specific T cell subsets or drive expansion of the corresponding T cell compartments by shaping the innate immune responses, which in turn regulates the process of Mtb infection.

## Discussion

By employing IL-37-Tg mice, the suppressive properties of IL-37 on inflammation have been consistently demonstrated [12]. Though IL-37 was implicated to be associated with TB [13,14], the exact role of IL-37 in the process of Mtb infection has not been elucidated. By generating IL-37-Tg mice, our data supports a protective role of IL-37 in mycobacterial infection by dampening overt inflammation and tissue impairment. On one side, IL-37 may control the exacerbated inflammation by downregulating the production of proinflammatory cytokines as well as reducing Th17 cell response, which thereby protect the host from excessive tissue damage at the site of infection [18]. On the other side, IL-37 enhances adaptive immunity exhibiting elevated Th1 cells which is critical for the containment of Mtb in IFN γ-dependent manner [18]. In accordance with the protective effect of IL-37 observed in the transgenic mice, we also demonstrated that polymorphism of IL-37 is associated with the susceptibility to PTB. Moreover, elevated abundance of IL-37 in TB patients was observed, which implied a positive feedback on the expression of IL-37 in response to Mtb infection.

IL-37 exerts its function in both intracellular and extracellular circumstances. Extracellular anti-inflammatory function of IL-37 was supported by either rIL-37 protein treatment or administration of neutralizing antibodies against IL-37 [19]. Moreover, recruitment of decoy IL-1R8 (SIGIRR) to IL-18Rα/IL-37 complex was demonstrated to be critical for anti-inflammatory function of extracellular IL-37 [15,20]. On the other way, endogenous IL-37 inhibits LPS-induced inflammatory response by association with signal transducer and transcriptional modulator Smad3 [12], a process indispensable of its nuclear translocation via caspase-1-mediated cleavage [21]. The protective effect of IL-37 against mycobacterial infection observed in transgenic mice can be conferred by either extracellular or intracellular IL-37. Provided that extracellular IL-37 is sufficient to protect Mtb infection as observed in IL-37-Tg mice, it would be of great potential to translate the recombinant protein into clinical therapeutic agents.

It has been reported that ManLAM induced IL-37 production through TLR2/p38 or ERK1/2 pathway in human alveolar epithelial cells [13]. Here we demonstrated that Mtb induced IL-37 production in macrophages as well. The production of IL-37 significantly inhibits the production of proinflammatory cytokines including IL-6 and TNF-α in Mtb-infected macrophages and mice, which reinforced that the inflammatory responses to MTB infection in tightly regulated. It still awaits further study to dissect the mechanism underlying the regulation of IL-37 production in response to Mtb infection.

To the best of our knowledge, our findings firstly demonstrated a close association of *IL-37* genetic variant rs3811047 G>A with TB susceptibility in Chinese population. Previous clinical studies have implicated that *IL-37* SNP rs3811047 associated with the susceptibility to gastric cardiac adenocarcinoma [22], autoimmune diseases including autoimmune thyroid disease (AITD) [23], ankylosing spondylitis (AS) [24] and disease activity of rheumatoid arthritis (RA) [25,26]. The variant rs3811047 is located at the exon of this gene, while the G>A allele leads to a mutation from alanine to threonine and confers resistance to Mtb infection. We speculate that the allele mutation may result in expressed products with altered structure and binding affinity to its receptors or partner proteins, which eventually affect TB susceptibility. However, the exact regulatory mechanism warrants further in-depth investigation.

IL-37 has been reported to exert a potent immunosuppressive role in the pathogenesis of human rheumatoid arthritis (RA) and in mice with collagen-induced arthritis (CIA) models by downregulating IL-17 and impairing of Th17 cell proliferation [27]. Consistently, less frequency of CD4^+^IL-17^+^Th17 cells in the spleen was observed in IL-37-Tg mice infected with BCG, which may correlate with the reduced pathological impairment in the lung. The expression of IL-37 in dendritic cells (DCs) was reported to inhibit adaptive immune response by promoting generation of semimature tolerogenic DCs [11]. Moreover, IL-37 expression in human CD4^+^CD25^+^Treg cells was demonstrated to promote the suppressive effect on T cell activation *in vitro* by upregulating secretory cytokines including TGF-β and IL-10, as well as expression of cell surface molecules including transcription factor Foxp3 and cytotoxic T-lymphocyte associated antigen (CTLA)-4 [16]. Here we monitored the effect of IL-37 on Treg response in the scenario of mycobacteria infection *in vivo* for the first time. Unexpectedly, a reduced frequency of CD4^+^CD25^+^Foxp3^+^Treg cells was observed in IL-37-Tg mice infected with BCG, which indicated that regulatory function of IL-37 on Treg cells is context-dependent. It’s also likely that IL-37 is involved in the regulation of the differentiation or proliferation of Treg cells. Furthermore, we observed a Th1 expansion in the spleen of IL-37-Tg mice infected with BCG. The expression of IL-37 in CD4+ T cells was reported previously [16], therefore it’s potentially interesting to investigate its direct effect on the differentiation of Th1 cells and the precise underlying mechanism.

In summary, in current study we demonstrated an association of *IL-37* gene polymorphism with TB susceptibility. IL-37 is significantly elevated in TB patients. IL-37 confers resistance to Mtb infection which possibly involves suppressing detrimental inflammation and modulating T cell responses. Therefore, IL-37 could serve as a novel therapeutic target and diagnosis marker for TB.

## Materials and Methods

### Study population and SNP genotyping

A case-control study was used to investigate the association between *IL-37* gene polymorphism and PTB susceptibility. The genome DNA extracted from 375 healthy controls and 435 PTB patients used for our previous *EBI3* polymorphism study [28] was applied for current study. We selected 3 SNPs for *IL-37* (rs2723176, rs3811047, rs6717710). The tag SNPs (rs2723176, rs3811047) were selected from the Phase 1 & 2 full dataset of the International HapMap Project and searched in region on chromosome 2q12 between 5 kb upstream and 2 kb downstream of *IL-37*. For tagged SNPs with an R^2^ linkage disequilibrium of >0.8 and minor allele frequency of >0.1. The SNP rs6717710 was located at 5' near gene region of *IL-37*. A custom-by-design 2×48-Plex SNPscan^™^ Kit (Cat#: G0104K; Genesky Biotechnologies Inc., Shanghai, China) was applied into genotyping as described previously [29].

### Cell culture, mouse peritoneal macrophage and human PBMC isolation

RPMI 1640 medium, FBS, penicillin, and streptomycin were purchased from Invitrogen (Shanghai, China). THP-1 cells were cultured with RPMI 1640 complete medium and differentiated with PMA (50 ng/ml) for further experiment. Mouse peritoneal macrophages were purified as previously described [30]. All cells were cultured at 37°C in a humidified incubator with 5% CO_2_.

For human PBMC isolation, 20 ml acid-citrate dextrose-treated blood was obtained from heathy donors (n = 7) and TB patients (n = 7). PBMC were isolated by centrifugation over lymphocyte separation medium. After three washes in phosphate-buffered saline (PBS), the PBMC were harvested and counted for further experiments. The human participants were recruited from March 5 to April 19 in 2014. The authors are not blind to the information of the individual participants. All protocols of the clinical study involved human participants were approved by the ethics committee of Department of Medicine and Life Science, Tongji University (permit number: 2012-FK-01) and the signed informed consent was obtained from all participants.

### Bacteria culture

*M*. *bovis* BCG and Mtb H37Rv were grown at 37°C with shaking in Middlebrook 7H9 broth (Becton Dickinson, Cockeysville, MD) with 0.05% Tween-80 and 10% oleic acid-albumin-dextrose-catalase (OADC) enrichment (Becton Dickinson, Sparks, MD). The single bacterial suspension was prepared as described previously [30].

### Immunoblot analysis

Cells were lysed in RIPA lysis buffer for 30 min on ice and cleared by centrifugation at full speed. The supernatants were boiled in SDS loading buffer. Equivalent amounts of proteins were separated by SDS-PAGE under reducing conditions and were transferred to polyvinylidene fluoride (PVDF) membrane, followed by immunoblotting with corresponding antibodies and horseradish peroxidase (HRP)-conjugated anti-rabbit antibodies. ECL reagent (Thermo Scientific) was applied for immunoblot analysis. Antibodies against IL-37 was purchase from Abcam (ab116282) and β-actin antibody was obtained from Cell Signaling Technology (Beverly, MA) and used for Western blot at a dilution of 1:1000.

### Intranasal infection of mice with BCG

IL-37 transgenic mouse with C57BL/6 background were generated. C57/BL6 mice were used as controls. All female mice were maintained in animal facility of Tongji University (Shanghai, China) and used at the age of 6 to 10 weeks. Mice were anesthetized by injection of a mixture of Ketamine (Sigma Chemical Co.) and were then infected i.n. with 4×10^7^ viable CFUs of BCG or with PBS as control. The animal protocol was approved by the Committee on the Ethics of Animal Experiments of the Shanghai Pulmonary Hospital affiliated to Tongji University (Permit Number: 2013–003). All surgery was performed under anesthesia by injection of a mixture of Ketamine (Sigma Chemical Co.) and all efforts were made to minimize suffering.

### Histological analysis

The lower left lobe of lung tissues dissected from infected or control mice were fixed in 4% PFA overnight and embedded in paraffin wax. Serial sections with the thickness of 2–3 μm were cut and five non-continuous sections from each mouse were stained with hematoxylin and eosin using standard histological protocols. The stained sections were visualized by light microscopy.

### Enumeration of BCG in organs of infected mice

The right lung tissues recovered from infected mice were homogenized in sterile PBS containing 0.1% TritonX-100 (SigmaAldrich). Numbers of viable mycobacteria in the lung were determined by plating 10-fold serial dilutions of lung homogenates on 7H11 agar plates. Colonies were counted after incubation at 37°C for 3 weeks.

### Intracellular survival of MTB in macrophages

Mouse peritoneal macrophages from WT and IL-37-Tg mice were infected with Mtb H37Rv for 1 h at MOI = 1, followed by wash and culture for indicated time. To assess the extracellular effect of IL-37 on the intracellular survival of Mtb, cells were cultured in the absence or presence of different concentrations of recombinant IL-37 (Abcam, ab153288) after washing the extracellular bacteria. Cells were then lysed with 0.1% Triton X-100 and plated in 10-fold serial dilutions on 7H11 agar plates. Colonies were counted after incubation at 37°C for 3 weeks.

### Spleen cell staining and flow cytometric analysis

Spleens were isolated, ground, and then filtered through a nylon sieve. Filtered cells were centrifuged at 1 000 *g* for 5 min. Single cells were suspended in red blood cell (RBC) lysis buffer to remove RBCs and were then cultured with RPMI 1640 complete medium for 16 h in the presence of protein transporter inhibitor. Cells were then harvested, washed twice with PBS and fixed with 4% PFA. Cell aliquots containing 5×10^5^ cells were suspended in 100 μl of staining buffer, mixed with 1 μl of the corresponding fluorescent antibodies against surface markers, and then incubated on ice for 20 min. Cells were then washed with PBS twice and permeabilized for staining of intracellular markers. Flow cytometric analysis of cell surface markers included CD3, CD4, CD25 and intracellular molecules including IFN γ, Foxp3, IL-17. PE, FITC, or APC labeled CD3, CD4, CD25, IFN γ, Foxp3 and IL-17 antibodies, as well as corresponding isotype control antibodies, were all purchased from eBioscience (San Diego, CA). Samples were run in a flow cytometer (BD Accuri C6) and subsequently analyzed with BD CFlow Plus software.

### ELISA

Peritoneal macrophages were infected with Mtb H37Rv at MOI 5 otherwise indicated for indicated times and the supernatants were harvested for quantification of cytokines released. The upper left lobe of lung tissues from mice infected with BCG were homogenized and centrifuged, and the supernatants were harvested for cytokines measurement. The peripheral blood was collected from active TB patients and healthy donors and sera were prepared for cytokines detection. The samples were collected from January to May in 2013 and the authors are not blind to the information of individual participants. ELISA kits to detect IL-37, IL-6 and TNF-α were all purchased from Raybiotech Inc., and the measurement was conducted according to the manufacturer’s instructions.

### RNA preparation, reverse transcription PCR, and quantitative real-time PCR

RNA was isolated using TRIzol reagent (Invitrogen) following the manufacturer’s instructions. Lung tissues were cut into pieces and vortexed with glass beads before the addition of TRIzol. Reverse transcription was done with iScript Reverse Transcription Supermix for RT-qPCR (Bio-Rad). Primers for quantitative real-time PCR (qRT-PCR) analysis is: IL-37_F: TTCTTTGCATTAGCCTCATCCTT, IL-37_R: CGTGCTGATTCCTTTTGGGC; GAPDH_F: AGGTCGGTGTGAACGGATTTG, GAPDH_R: TGTAGACCATGTAGTTGAGGTCA. qRT-PCR was run on an Applied Biosystems 7300 Real-time PCR system with SYBR^®^ Green Real-Time PCR Master Mixes (TOYOBO). All PCR samples were analyzed in triplicate within each experiment, and all experiments were replicated for at least three times.

### Statistical analysis

For SNP analysis, Hardy-Weinberg equilibrium of each polymorphism was determined by the program HWE. The significant differences of allele and genotype frequencies were calculated using Pearson’s χ2 test. The genetic association analyses were performed using the SPSS (SPSS Inc, Chicago, IL).

Data are expressed as mean±SEM unless otherwise indicated. GraphPad Prism5 (GraphPad Software) was used for statistical analysis. Either Student’s *t* test or a two way ANOVA followed by a Bonferroni’s post test as indicated was applied to analyze the differences between groups. Bacterial titers were analyzed using the Mann-Whitney *U* test. Values of *p* < 0.05 represented a statistically significant difference.

## Supporting Information

S1 Fig Construction of IL-37-Tg mouse. **(A)** Vector map of pBROAD3-IL-37. **(B)** Primers designed for the identification of IL-37-Tg mice by PCR. **(C)** Western blot detection of IL-37 expression in the lysates of peritoneal macrophages isolated from WT and IL-37-Tg mice.
(TIF)

S2 Fig T cell responses in IL-37 transgenic mice during mycobacterial infection. Kinetics of T cells **(A)**, CD4^+^ T cells **(B)** and CD8^+^ T cells **(C)** in the spleens of WT and IL-37-Tg mice infected with BCG. The frequencies of CD3^+^ **(A)**, CD3^+^CD4^+^ **(B)** and CD3^+^CD8^+^ **(C)** cells among gated lymphocytes were determined at indicated times p.i. Results are the mean±SEM and are pooled from two independent experiments; two way ANOVA with Bonferroni’s post test. *, p<0.05.
(TIF)
