# Supplementary figures and images for: IL-37 Confers Protection against Mycobacterial Infection Involving Suppressing Inflammation and Modulating T Cell Activation
Source: PLoS One. 2017 Jan 11;12(1):e0169922. doi: 10.1371/journal.pone.0169922 (PMC5226736; doi:10.1371/journal.pone.0169922)

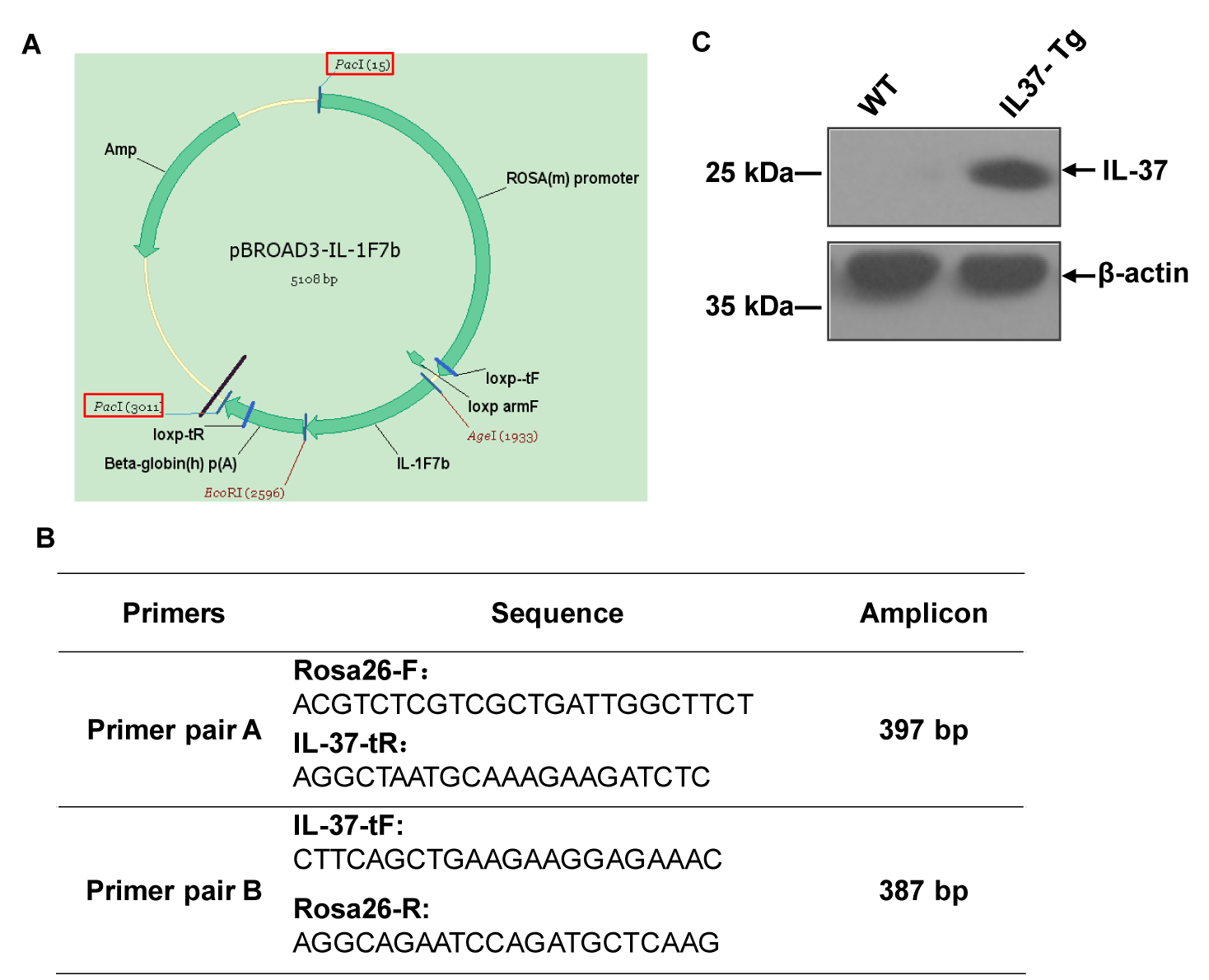

Supplement: S1 Fig — (A) Vector map of pBROAD3-IL-37. (B) Primers designed for the identification of IL-37-Tg mice by PCR. (C) Western blot detection of IL-37 expression in the lysates of peritoneal macrophages isolated from WT and IL-37-Tg mice. (TIF) [file pone.0169922.s001.tif]

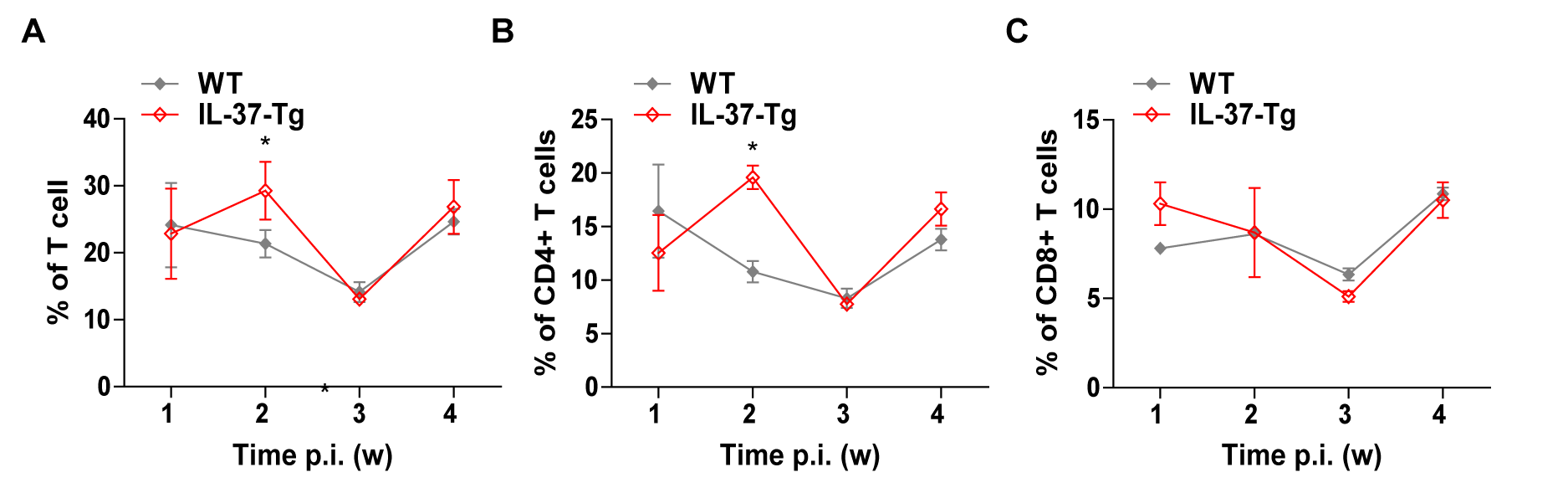

Supplement: S2 Fig — Kinetics of T cells (A), CD4+ T cells (B) and CD8+ T cells (C) in the spleens of WT and IL-37-Tg mice infected with BCG. The frequencies of CD3+ (A), CD3+CD4+ (B) and CD3+CD8+ (C) cells among gated lymphocytes were determined at indicated times p.i. Results are the mean±SEM and are pooled from two independent experiments; two way ANOVA with Bonferroni’s post test. *, p<0.05. (TIF) [file pone.0169922.s002.tif]
